# The Occurrence of *Klebsiella pneumoniae* in Drainage Fluid After Pancreaticoduodenectomy: Risk Factors and Clinical Impacts

**DOI:** 10.3389/fmicb.2021.763296

**Published:** 2021-10-26

**Authors:** Yifei Yang, Xu Fu, Zhenghua Cai, Yudong Qiu, Liang Mao

**Affiliations:** ^1^Department of Hepatopancreatobiliary Surgery, Nanjing Drum Tower Hospital, The Affiliated Hospital of Nanjing University Medical School, Nanjing, China; ^2^Department of Hepatopancreatobiliary Surgery, Nanjing Drum Tower Hospital Clinical College of Nanjing Medical University, Nanjing, China

**Keywords:** *Klebsiella pneumoniae*, pancreaticoduodenectomy, risk factor, postoperative complication, clinical impact

## Abstract

To investigate the risk factors and clinical impacts of the occurrence of *Klebsiella pneumoniae* isolated from drainage fluid in patients undergoing pancreaticoduodenectomy (PD). Clinicopathological data of all patients who underwent PD from January 2018 to March 2021 were analyzed retrospectively. The univariate and multivariate analyses were performed to identify independent risk factors for the occurrence of *K. pneumoniae* in drainage fluid and its clinical impacts on postoperative complications. Of the included 284 patients, 49 (17.2%) patients isolated *K. pneumoniae* in drain samples after PD. Preoperative biliary drainage (OR = 1.962, *p* = 0.037) independently predicted the contamination of *K. pneumoniae* in drain samples after PD. The rate of clinically relevant postoperative pancreatic fistula (CR-POPF), major complications (Clavien–Dindo Grade ≥ III), post-pancreatectomy hemorrhage (PPH), organ/space surgical site infection (SSI), and biliary leakage (BL) were significantly higher in *K. pneumoniae* positive group both in the univariate and multivariate analyses. Preventive measures and treatments for combating *K. pneumoniae* contamination may be beneficial to the perioperative outcomes of patients after PD.

## Introduction

Pancreaticoduodenectomy (PD) is one of the most complex operations performed for diseases localized in the periampullary region. Despite significant progress in surgical techniques and perioperative management, the postoperative morbidity rate remains high at 64% (Satoi et al., 2017). A number of studies have shown that bacterial contamination of drain fluid adversely affects the development of postoperative complications (Sugiura et al., 2015; Sato et al., 2017; Yang et al., 2018). *Klebsiella pneumoniae* is a prominent opportunistic pathogen, found in the oral cavity, skin, biliary tract, and intestines, inducing types of hospital-acquired infections (Liu et al., 2020). *K. pneumoniae* tops the list of multidrug-resistant pathogens focusing on the development of new treatments published by WHO (Baraldi et al., 2018). However, rare studies demonstrated the relationship between the occurrence of *K. pneumoniae* in drain samples and postoperative complications in patients undergoing PD.

Therefore, the main emphasis of this paper was placed on the clinical impacts of *K. pneumoniae* in drainage fluid after PD. Furthermore, we explored the risk factors about the occurrence of *K. pneumoniae*.

## Materials and Methods

### Patients and Data Collection

Retrospective analysis was conducted on 284 eligible patients who underwent PD from January 2018 to March 2021 at the Department of Hepatopancreatobiliary Surgery, Nanjing Drum Tower Hospital, The Affiliated Hospital of Medical School of Nanjing University. The inclusion criteria were as follows: (1) operation in the form of conventional PD or pylorus-preserving PD (PPPD); (2) no evidence of distant metastasis at the time of diagnosis; (3) > 18 years of age; and (4) at least one drain sample routinely sent for culture after operation. The exclusion criteria were as follows: (1) undergone concomitant hepatic or colon resection; (2) history of neoadjuvant chemotherapy; and (3) clinical data were incomplete.

The clinical data collected from medical records included age, sex, hypertension, diabetes mellitus, smoking, drinking, body mass index (BMI), preoperative jaundice, preoperative biliary drainage (PBD), history of abdominal surgery, preoperative laboratory data (e.g., total bilirubin, albumin, C-reactive protein, hemoglobin of peripheral blood), intraoperative parameters (e.g., type of resection, vessel resection, size of pancreatic duct, pancreatic consistency, operation time, volume of blood loss, and transfusion), pathological diagnosis, postoperative complications, and microorganisms cultured from drain samples after PD. For the outcome analysis, only the 11 most frequently detected bacterial strains as well as the 9 most common postoperative complications were included. Furthermore, all patients were assigned to two groups according to the occurrence of *K. pneumoniae* in drain fluid: *K. pneumoniae* positive group and *K. pneumoniae* negative group including patients with all other microorganisms.

### Surgical Procedures and Perioperative Management

Reconstruction of the digestive tract was accomplished by the modified Child’s method. Pancreatojejunostomy (PJ) was performed with a manual duct-to-mucosal, end-to-side, double-layer interrupted anastomosis method. Hepaticojejunostomy (HJ) was completed by manual end-to-side, monolayer continuous suture anastomosis on the same jejunal loop. The internal unabsorbed pancreatic duct drainage tube was routinely inserted according to the diameter of main pancreatic duct. Finally, two closed-suction peritoneal drainage tubes were routinely placed at the superior and inferior sides of PJ.

Standard perioperative management was applied for all patients. PBD by means of endoscopic nasobiliary drainage or percutaneous transhepatic cholangial drainage was received in the following situations: hyperbilirubinemia with total bilirubin level ≥ 15 mg/dl (>258 μmol/L), preoperative cholangitis occurred, or jaundice with malnutrition (Fang et al., 2012; Iacono et al., 2013). For patients with preoperative cholangitis, it was addressed in accordance with the Tokyo Guidelines 2018 for the management of acute cholangitis/acute cholecystitis before surgery (Takada, 2018). All patients received routine intravenous prophylactic antibiotics from at least 30 min before surgery to 48 h after surgery. The choice of antibiotic differed among the patients: routinely a third-generation cephalosporin (Ceftriaxone) or amikacin (Bratzler et al., 2013) in case of allergy to cephalosporin in the non-PBD patients or PBD patients with positive biliary drainage cultures susceptible to Ceftriaxone. In PBD patients with Ceftriaxone resistance biliary drainage cultures, the prophylactic antibiotics were selected based on the antimicrobial susceptibility (Gomi et al., 2018). Somatostatin analogs were used routinely until postoperative day (POD) 7.

Drain amylase concentration, and bacterial smear and culture test for aerobic/anaerobic microorganisms were measured routinely from each of the peritoneal drains on PODs 1, 3, 5, and 7 and every 2 to 3 days thereafter until drains were removed. The peritoneal drainage tubes were removed on POD 7 after the abdominal enhanced CT conducted on POD 7 showed no evidence of CR-POPF or fluid collection. In the patients who underwent CR-POPF or fluid accumulation on CT, the drains were retained until the CR-POPF healed, additional surgical drainage was performed under ultrasonographic guidance, and broad-spectrum antibiotic therapy was initiated.

### Definition of Variables

Postoperative complications were classified according to the Clavien–Dindo classification, with major complications defined as grade ≥ III (Dindo et al., 2004). The definition and grading system of POPF was defined according to the International Study Group of Pancreatic Fistula guidelines (Bassi et al., 2017). Diagnostic criteria of biliary leakage (BL) were based on the guidelines of the International Study Group of Liver Surgery (Koch et al., 2011). Chyle leakage, post-pancreatectomy hemorrhage (PPH), and delayed gastric emptying were defined according to the International Study Group of Pancreatic Surgery (Wente et al., 2007a,b; Besselink et al., 2017). Surgical site infections (SSIs), included incisional SSI and organ/space SSI, were diagnosed according to the criteria established by the Center for Disease Control (Mangram et al., 1999). According to the magnitude of postoperative complications, we defined major complications (Clavien–Dindo ≥ III), CR-POPF, organ/space SSI, PPH, and BL as severe complications.

### Statistics

Statistical analyses were completed by SPSS 23.0 software for Windows. Categorical variables were compared using χ*^2^* test or Fisher’s exact test as appropriated, expressed as absolute number and percentage. Continuous variables were compared with independent-samples *t-*test, which was described as mean ± SD when the data showed normal distribution. Mann–Whitney *U* test was used and expressed as median (interquartile range) when they were not normally distributed. Variables with *p* < 0.1 in univariate analysis entered the multivariate logistic regression model with a stepwise forward approach. Odds ratio (OR) and 95% CIs were obtained. A *p*-value of <0.05, two sides, was considered as statistically significant.

## Results

### Patient Characteristics and Bacteriology

The demographics of all 284 patients are described in Table 1. The group consisted of 174 (61.3%) men and 110 (38.7%) women, and the median age was 64.0 (55.2–70.0) years. Pancreatic ductal adenocarcinoma accounted for 23.6% in the group. PPPD was performed in 75 (31.9%) patients. In total, 86 (30.3%) patients performed PBD.

**TABLE 1:** Patients characteristics.

**Characteristics**	**All patients (*n* = 284)**
Age (median, IQR), years	64.0 (55.2–70.0)
BMI (mean ± SD), kg/m^2^	23.4 ± 3.6
**Gender, n (%)**	
Male	174 (61.3)
Female	110 (38.7)
Hypertension, n (%)	98 (34.5)
DM, n (%)	52 (18.1)
Smoking, n (%)	69 (24.3)
Drinking, n (%)	49 (17.3)
Abdominal surgery history, n (%)	86 (30.3)
Preoperative jaundice, n (%)	111 (39.1)
PBD, n (%)	86 (30.3)
TB (median, IQR), μmol/L	15.9 (9.2–58.8)
Alb (mean ± SD), g/L	38.8 ± 3.1
CRP (median, IQR), mg/L	4.5 (2.9–6.7)
Hemoglobin (mean ± SD), g/L	123.1 ± 18.0
**Pathology, n (%)**	
PDAC	67 (23.6)
Others	217 (76.4)
**Type of resection, n (%)**	
PD	196 (69.0)
PPPD	88 (31.0)
**Vessel resection, n (%)**	
Yes	11 (3.8)
No	273 (96.2)
**Pancreatic consistency, n (%)**	
Hard	40 (14.1)
Soft	244 (85.9)
Diameter of MPD (median, IQR), mm	3.0 (2.0–5.0)
Operation time (median, IQR), min	370.0 (306.3–435.0)
Blood loss volume (median, IQR), ml	400.0 (300.0–637.5)
Blood transfusion (median, IQR), ml	0.0 (0.0–675.0)

*IQR, interquartile range; SD, standard deviation; BMI, body mass index; DM, diabetes mellitus; PBD, preoperative biliary drainage, TB, total bilirubin; Alb, albumin; PD, pancreaticoduodenectomy; PPPD, pylorus-preserving pancreaticoduodenectomy; MPD, main pancreatic duct; PDAC, pancreatic ductal adenocarcinoma; CRP, C-reactive protein.*

The microorganisms cultured from drain fluid are listed in Table 2. The most common bacterial species isolated were *K. pneumoniae* (*n* = 49, 17.3%), followed by *E. faecalis* (*n* = 41, 14.4%), *S. epidermidis* (*n* = 29, 10.2%), *E. faecium* (*n* = 26, 9.1%), *S. haemolyticus* (*n* = 25, 8.9%), and *E. coli* (*n* = 24, 8.5%). Polymicrobial mixed flora were detected in 117 (41.1%) patients.

**TABLE 2:** Microorganisms cultured from drainage fluid after pancreaticoduodenectomy.

**Microorganisms**	**All patients (*n* = 284)**
*K. pneumoniae*, n (%)	49 (17.3)
*E. faecalis*, n (%)	41 (14.4)
*S. epidermidis*, n (%)	29 (10.2)
*E. faecium*, n (%)	26 (9.1)
*S. haemolyticus*, n (%)	25 (8.9)
*E. coli*, n (%)	24 (8.5)
*Fungus*, n (%)	20 (7.0)
*E. cloacae*, n (%)	18 (6.3)
*P. aeruginosa*, n (%)	13 (4.6)
*A. baumannii*, n (%)	12 (4.2)
*S. aureus*, n (%)	10 (3.5)

### Perioperative Outcomes and Risk Factors for the Contamination of *Klebsiella pneumoniae*

The most common and hazardous postoperative complications are summarized in Table 3. The rate of CR-POPF was significantly higher in the *K. pneumoniae* positive group compared with the negative group (65.3 vs. 32.3%, *p* < 0.001). Biliary leakage was reported in 8 (16.3%) cases in the *K. pneumoniae* positive group and 12 (5.1%) in the negative group (*p* = 0.005). The percentage of patients with PPH and major complications (Clavien–Dindo grade ≥ III) was significantly higher in the *K. pneumoniae* positive group than the negative group (24.5 vs. 5.1%, *p* < 0.001; 40.8 vs. 17.0%, *p* < 0.001). Although the rate of incisional SSI was similar between both groups (6.1 vs. 5.5%, *p* = 0.870), the incidence of organ/space SSI was significantly higher in the *K. pneumoniae* positive group (61.2 vs. 22.9%, *p* < 0.001).

**TABLE 3:** Postoperative complications of all cohorts and stratified according to the contamination of *K. pneumoniae*.

**Complications**	**All patients (*n* = 284)**	***K. pneumoniae* negative (*n* = 235)**	***K. pneumoniae* positive (*n* = 49)**	***p* value**
**Severe complications**				
Pancreatic fistula, n (%)				<0.001
non-PF/biochemical fistula	176 (62.0)	159 (67.7)	17 (34.7)	
CR-POPF	108 (38.0)	76 (32.3)	32 (65.3)	
Major complication, n (%)	60 (21.1)	40 (17.0)	20 (40.8)	<0.001
PPH, n (%)	24 (8.4)	12 (5.1)	12 (24.5)	<0.001
BL, n (%)	20 (7.0)	12 (5.1)	8 (16.3)	0.005
Organ/space SSI, n (%)	84 (29.6)	54 (22.9)	30 (61.2)	<0.001
**Non-severe complication**				
Incisional SSI, n (%)	16 (5.6)	13 (5.5)	3 (6.1)	0.870
DGE, n (%)	96 (33.8)	82 (34.9)	14 (28.6)	0.395
CL, n (%)	35 (12.3)	32 (13.6)	3 (6.1)	0.147

*PF, pancreatic fistula; CR-POPF, clinically relevant postoperative pancreatic fistula (Grade B/C); BL, biliary leakage; CL, chyle leakage; PPH, post-pancreatectomy hemorrhage; DGE, delayed gastric emptying; SSI, surgical site infection.*

We further analyzed the risk factors of the *K. pneumoniae* contamination in drain samples. A total of 86 (30.3%) patients performed PBD, which was significantly higher in the *K. pneumoniae* positive group (48.7 vs. 27.7%, *p* = 0.035). Furthermore, PBD was the only independent risk factor of the *K. pneumoniae* contamination according to multivariate logistic regression analysis (Table 4).

**TABLE 4:** Univariate and multivariate analysis of the contamination of *K. pneumoniae* of drain fluid after PD.

**Variables**	***K. pneumoniae* negative (*n* = 235)**	***K. pneumoniae* positive (*n* = 49)**	***p***	**OR (95%CI)**	***p***
Age (median, IQR), years	63.0 (55.0–70.0)	65.0 (56.0–71.0)	0.428		
BMI (mean ± SD), kg/m^2^	23.4 ± 3.5	24.0 ± 2.1	0.101		
Gender, n (%)			0.200		
Male	140 (59.6)	34 (69.4)			
Female	95 (40.4)	15 (30.6)			
Hypertension, n (%)	81 (34.5)	17 (34.7)	0.976		
DM, n (%)	56 (23.8)	13 (26.5)	0.688		
Smoking, n (%)	46 (26.1)	23 (21.3)	0.356		
Drinking, n (%)	44 (18.7)	5 (10.2)	0.151		
Abdominal surgery history, n (%)	71 (30.2)	15 (30.6)	0.956		
Preoperative jaundice, n (%)	91 (38.7)	20 (40.8)	0.785		
PBD, n (%)	65 (27.7)	21 (48.7)	0.035	1.962 (1.041–3.697)	0.037
TB (median, IQR), μmol/L	15.8 (8.8–58.9)	16.6 (11.8–59.9)	0.329		
Alb (mean ± SD), g/L	38.8 ± 3.1	38.9 ± 3.3	0.896		
CRP (median, IQR), mg/L	4.5 (2.9–6.9)	4.3 (2.7–6.3)	0.537		
Hb (mean ± SD), g/L	123.4 ± 17.3	121.2 ± 21.3	0.581		
Pathology, n (%)			0.564		
PDAC	57 (24.3)	10 (20.4)			
Others	178 (75.7)	39 (79.6)			
Type of resection, n (%)			0.458		
PD	115 (68.1)	91 (73.5)			
PPPD	75 (31.9)	13 (26.5)			
Vessel resection, n (%)			0.370		
Yes	8 (3.4)	3 (6.1)			
No	227 (96.6)	46 (93.9)			
Pancreatic consistency, n (%)			0.684		
Hard	34 (14.5)	6 (12.2)			
Soft	201 (85.5)	43 (87.8)			
Diameter of MPD (median, IQR), mm	3.0 (2.0–5.0)	3.0 (2.0–4.0)	0.232		
Operation time (median, IQR), min	370.0 (300.0–440.0)	385.0 (332.5–432.5)	0.264		
Blood loss volume (median, IQR), ml	400.0 (300.0–600.0)	500.0 (250.0–800.0)	0.751		
Blood transfusion (median, IQR), ml	0.0 (0.0–600.0)	0.0 (0.0–937.5)	0.220		

*POD, postoperative day; IQR, interquartile range; SD, standard deviation; OR, odds ratio; CI, confidence interval; BMI, body mass index; DM, diabetes mellitus; PBD, preoperative biliary drainage; TB, total bilirubin, Hb, hemoglobin; Alb, albumin; PD, pancreaticoduodenectomy; PPPD, pylorus-preserving pancreaticoduodenectomy; MPD, main pancreatic duct; PDAC, pancreatic ductal adenocarcinoma; CRP, C-reactive protein.*

### Impact of *Klebsiella pneumoniae* Contamination on Perioperative Outcomes

Severe complications were related to the *K. pneumoniae* contamination identified by the univariate analysis (Table 3). To validate the critical role of the contamination of *K. pneumoniae* in drain fluid, we performed another analysis to identified the risk factors about the postoperative complications and only indicators with significant differences in multivariate analysis are listed in Tables 5, 6. *K. pneumoniae* contamination was identified as an independent risk factor for major complications (OR = 1.980, *p* = 0.048), CR-POPF (OR = 2.588, *p* = 0.011), organ/space SSI (OR = 3.162, *p* = 0.003), PPH (OR = 7.065, *p* < 0.001), and BL (OR = 4.302, *p* = 0.004) in multivariate analysis (Table 5). The polymicrobial mixed flora adversely affected the major complications (OR = 2.491, *p* = 0.014), CR-POPF (OR = 5.406, *p* < 0.001), organ/space SSI (OR = 5.396, *p* < 0.001), and incisional SSI (OR = 3.689, *p* < 0.020) (Tables 5, 6).

**TABLE 5:** Univariate and multivariate analysis for risk factors of sever complication.

**Variables**	**Major complications (*n* = 60)**			**CR-POPF (*n* = 108)**			**Organ/space SSI (*n* = 84)**			**PPH (*n* = 24)**			**BL (*n* = 20)**		
	**Univariate**	**Multivariate**		**Univariate**	**Multivariate**		**Univariate**	**Multivariate**		**Univariate**	**Multivariate**		**Univariate**	**Multivariate**	
		**OR (95%CI)**	***p***		**OR (95%CI)**	***p***		**OR (95%CI)**	***p***		**OR (95%CI)**	***p***		**OR (95%CI)**	***p***
BMI	0.523			0.078			0.240			0.763			0.377		
Gender	0.334			0.087			0.226			0.020			0.406		
Hypertension	0.026	1.883 (1.011–3.506)	0.046	0.337			0.414			0.223			0.660		
Abdominal surgery history	0.178			0.075			0.491			0.901			0.123		
Preoperative jaundice	0.644			0.051			0.267			0.252			0.006	0.133 (0.029–0.607)	0.009
PBD	0.126			0.253			<0.001	2.096 (1.112–3.916)	0.020	0.421			0.123		
TB	0.380			0.101			0.056			0.298			0.542		
Alb	0.798			0.885			0.060			0.612			0.126		
Hb	0.508			0.536			0.359			0.240			0.021		
Pathology	0.155			0.015			0.140			0.739			0.213		
Pancreatic consistency	0.063			0.001	0.207 (0.074–0.578)	0.003	0.950			0.816			0.226		
Diameter of MPD	0.892			0.008	0.863 (0.753–0.989)	0.035	0.155			0.687			0.770		
Operation time	0.583			0.245			0.008			0.307			0.685		
Blood transfusion	0.794			0.863			0.055			0.590			0.330		
*K. pneumoniae*	<0.001	2.688 (1.358–5.320)	0.005	<0.001	4.522 (2.237–9.143)	<0.001	<0.001	5.413 (2.605–11.246)	<0.001	<0.001	7.065 (2.945–16.948)	<0.001	0.005	4.302 (1.595–11.602)	0.004
*E. faecalis*	0.027			0.003	2.474 (1.138–5.379)	0.022	<0.001	4.398 (2.030–9.528)	<0.001	0.351			0.941		
*S. epidermidis*	0.048			0.991			0.298			0.08			0.534		
*E. faecium*	<0.001	3.955 (1.623–9.638)	0.002	<0.001	4.371 (1.510–12.654)		<0.001	4.610 (1.659–12.808)	0.003	0.182			0.011		
*S. haemolyticus*	0.378			0.832			0.856			0.402			0.534		
*E. coli*	0.313			0.010	3.611 (1.253–10.405)		<0.001	5.173 (1.945–13.758)	0.001	0.983			0.006	5.195 (1.539–17.540)	0.008
*Fungus*	0.001	3.323 (1.203–9.181)	0.021	0.002			0.010			0.275			0.592		
*E. cloacae*	0.906			0.010			0.013			0.195			0.799		
*P. aeruginosa*	0.024			0.018			0.050			0.358			0.021		
*A. baumannii*	0.075			0.139			0.113			0.296			0.858		
*S. aureus*	0.380			0.232			0.032	4.449 (1.003–19.741)	0.050	0.858			0.376		

*CR-POPF, clinically relevant postoperative pancreatic fistula (Grade B/C); BL, biliary leakage; PPH, post-pancreatectomy hemorrhage; SSI, surgical site infection; OR, odds ratio; CI, confidence interval; BMI, body mass index; PBD, preoperative biliary drainage; TB, total bilirubin; Hb, hemoglobin; Alb, albumin; MPD, main pancreatic duct.*

**TABLE 6:** Univariate and multivariate analysis for risk factors of non-sever complication.

**Variables**	**DGE (*n* = 60)**			**CL (*n* = 35)**			**Incisional SSI (*n* = 16)**		
	**Univariate analysis**	**Multivariate analysis**		**Univariate analysis**	**Multivariate analysis**		**Univariate analysis**	**Multivariate analysis**	
		**OR (95%CI)**	***p***		**OR (95%CI)**	***p***		**OR (95%CI)**	***p***
Age	0.043			0.147			0.678		
Smoking	0.206			0.006	2.836 (1.346–5.973)	0.006	0.946		
Drinking	0.237			0.058			0.127		
Preoperative jaundice	0.524			0.625			0.086		
PBD	0.266			0.084			0.301		
Alb	0.026	0.904 (0.829–0.985)	0.021	0.437			0.047	1.200 (1.009–1.426)	0.039
CRP	0.087			0.926			0.864		
Hb	0.070			0.537			0.056		
Type of resection	<0.001	0.213 (0.108–0.418)	<0.001	<0.001			0.276		
Vessel resection	0.077			0.124			0.612		
Diameter of MPD	0.030			0.878			0.764		
Operation time	0.023			0.053			0.540		
*S. epidermidis*	0.185			0.041	2.805 (1.074–7.326)	0.035	0.044	3.619 (1.057–12.387)	0.041
*S. aureus*	0.673			0.452			0.045		

*CL, chyle leakage; DGE, delayed gastric emptying; SSI, surgical site infection; OR, odds ratio; CI, confidence interval; PBD, preoperative biliary drainage; TB, total bilirubin; Hb, hemoglobin; Alb, albumin; MPD, main pancreatic duct.*

## Discussion

The current study was designed to invest risk factors and clinical impacts of *K. pneumoniae* contamination in drain fluid after PD on postoperative complications. It revealed that *K. pneumoniae* was the most frequently detected microorganism and increased several types of complications, including CR-POPF, major complications, PPH, BL, and organ/space SSI. In addition, PBD was identified as the only independent risk factor of the *K. pneumoniae* contamination in drain samples.

*Klebsiella pneumoniae* is a frequently gram-negative opportunistic pathogen, mainly concentrated in the gastrointestinal tract. Based on our findings, the *K. pneumoniae* contamination in drain fluid was correlated with PBD. Interestingly, the association between PBD and postoperative complications was not significant, except for organ/space SSI. The relationship between PBD and organ/space SSI was reported previously (Ngu et al., 2013). Gu et al. (2020) demonstrated that the history of biliary surgery, including endoscopic retrograde cholangiopancreatography, was a risk factor of the contamination of bile by *K. pneumoniae*. The surgical intervention destroyed the normal structure of Oddi sphincter, leading to the reflux from the gastrointestinal tract into the biliary tract and pancreas (Limongelli et al., 2007). *K. pneumoniae* can form biofilm, which leads to colonization of bile, biliary stent, and gastrointestinal tract (Wang G. et al., 2020). Colonized *K. pneumoniae* can result in the contamination of surgical wound and peritoneal cavity. For the purpose of reducing the rate of *K. pneumoniae* contamination, PBD indications should be controlled strictly and performed only in selected patients. At the same time, this finding emphasizes the significance of routine bile culture both during biliary drainage and intraoperatively, especially if *K. pneumoniae* is colonized.

*Klebsiella pneumoniae* had the highest incidence in our study, causing postoperative complications more frequently compared with other gram-negative/positive opportunistic pathogens, and the major microorganisms isolated from drain samples were comparable with previous studies (Behrman and Zarzaur, 2008; Sugiura et al., 2012; Liu et al., 2020). Polymicrobial mixed flora affected four types of complications less than the occurrence of *K. pneumoniae*. Some investigators termed these to several possibilities: (1) naturally resist antibiotics (Holt et al., 2015), (2) competitive advantage with other bacteria (Struve et al., 2015; Hsieh et al., 2019), (3) acquire a wide range of antibiotics resistance and virulence-enhancing genes (Wyres et al., 2019), and (4) formation of biofilm (Wang G. et al., 2020). The increased virulence of *K. pneumonia* accompanies production of lipopolysaccharide (LPS) which has been proved to activate the trypsinogen in pancreatic juice (Traverso and MacFarlane, 1987; Vaccaro et al., 1996). Increased virulence with production of LPS, formation of the biofilm, and resistance to β-lactam antibiotics prompt the prevalence of *K. pneumonia*, induce the colonization of *K. pneumonia* adjacent to the anastomosis, activate the trypsinogen, and erode the surrounding tissues. Acquisition of hypervirulence, antibiotic resistance, and formation of biofilm make the universal treatment with a third-generation cephalosporin less effective and increase the development of postoperative complications including CR-POPF, organ/space SSI, major complications, PPH, and biliary leakage.

Rogers et al. (2017) found an increased abundance of *K. pneumoniae* in pancreas, bile, and jejunal and fecal samples of PD patients, particularly those who develop POPF. In fact, most clinicians intervene the treatment based on clinical symptoms but not culture results, which cannot be fully used to guide therapy. A standard intravenous antibiotic prophylaxis regimen consisting with a third-generation cephalosporin was performed to patients prior to and after surgery. However, the acquisition of hypervirulence and antimicrobial resistance of *K. pneumoniae* always lead to the routine antibiotic prophylaxis regimen failure. At present, in the clinical treatment of *K. pneumoniae* infection, a combination of tigecycline, colistin, and meropenem improves the outcomes of patients (Kaur et al., 2018). The study conducted by Wang Q. et al. (2020) identified that a combination of sensitive antibiotics according to the susceptibility test with extension of the infusion time and increasing the dose in the allowable range can improve the outcomes of patients. Thus, it is important to conduct drain fluid culture test and early detect the occurrence of *K. pneumoniae* for adjusting routine antibiotics strategy of postoperative period. However, the exact indication and timing of the readjusting of antibiotic regimen needs further investigation.

There are some weaknesses in the present study. First, because of its retrospective study design, there were some inevitable variances in selection and patient management bias, resulting in diagnostic bias. Second, the sample size was small and all patients were from a single center, implying an insufficient statistical efficiency.

In conclusion, our study identified that the contamination of *K. pneumoniae* of drain fluid after PD had a negative impact of postoperative complications. Furthermore, *K. pneumoniae* contamination was significantly related to PBD. To reduce the incidence of *K. pneumoniae* contamination, PBD should be controlled strictly and performed only in selected patients. Bile culture during biliary drainage or operation, and drainage culture test after PD should be performed routinely for the early detection of the *K. pneumoniae* contamination. The results in this study imply that patients may benefit from antibiotic prophylaxis to combat *K. pneumoniae* contamination, although further study is needed.

## Data Availability Statement

The original contributions presented in the study are included in the article/supplementary material, further inquiries can be directed to the corresponding author.

## Ethics Statement

This study protocol was reviewed and approved by the Health Research Ethics Board of Drum Tower Hospital of Nanjing University Medical School (approval no. 2021-271-01).

## Author Contributions

LM and YQ: study concept and design. YY, ZC, and XF: data acquisition. YY and XF: data analysis and interpretation. YY: drafting of the manuscript and statistical analysis. LM: critical revision of the manuscript for important intellectual content. All authors contributed to the article and approved the submitted version.

## Conflict of Interest

The authors declare that the research was conducted in the absence of any commercial or financial relationships that could be construed as a potential conflict of interest.

## Publisher’s Note

All claims expressed in this article are solely those of the authors and do not necessarily represent those of their affiliated organizations, or those of the publisher, the editors and the reviewers. Any product that may be evaluated in this article, or claim that may be made by its manufacturer, is not guaranteed or endorsed by the publisher.
